# The role of governance in shaping health system reform: a case study of the design and implementation of new health regions in Ireland, 2018–2023

**DOI:** 10.1186/s12913-024-11048-2

**Published:** 2024-05-03

**Authors:** Katharine Schulmann, Carlos Bruen, Sarah Parker, Rikke Siersbaek, Luisne Mac Conghail, Sara Burke

**Affiliations:** 1 Centre for Health Policy & Management, Trinity College Dublin, Dublin, D02 YT92 Ireland; 2 Discipline of Clinical Medicine, Trinity College Dublin, Dublin, D08 W9RT Ireland

**Keywords:** Health system reform, Health governance, Healthcare decentralisation

## Abstract

**Background:**

Effective governance arrangements are central to the successful functioning of health systems. While the significance of governance as a concept is acknowledged within health systems research, its interplay with health system reform initiatives remains underexplored in the literature. This study focuses on the development of new regional health structures in Ireland in the period 2018–2023, one part of a broader health system reform programme aimed at greater universalism, in order to scrutinise how aspects of governance impact on the reform process, from policy design through to implementation.

**Methods:**

This qualitative, multi-method study draws on document analysis of official documents relevant to the reform process, as well as twelve semi-structured interviews with key informants from across the health sector. Interviews were analysed according to thematic analysis methodology. Conceiving governance as comprising five domains (Transparency, Accountability, Participation, Integrity, Capacity) the research uses the TAPIC framework for health governance as a conceptual starting point and as initial, deductive analytic categories for data analysis.

**Results:**

The analysis reveals important lessons for policymakers across the five TAPIC domains of governance. These include deficiencies in accountability arrangements, poor transparency within the system and vis-à-vis external stakeholders and the public, and periods during which a lack of clarity in terms of roles and responsibilities for various process and key decisions related to the reform were identified. Inadequate resourcing of implementation capacity, competing policy visions and changing decision-making arrangements, among others, were found to have originated in and continuously reproduced a lack of trust between key institutional actors. The findings highlight how these challenges can be addressed through strengthening governance arrangements and processes. Importantly, the research reveals the interwoven nature of the five TAPIC dimensions of governance and the need to engage with the complexity and relationality of health system reform processes.

**Conclusions:**

Large scale health system reform is a complex process and its governance presents distinct challenges and opportunities for stakeholders. To understand and be able to address these, and to move beyond formulaic prescriptions, critical analysis of the historical context surrounding the policy reform and the institutional relationships at its core are needed.

**Supplementary Information:**

The online version contains supplementary material available at 10.1186/s12913-024-11048-2.

## Introduction

Ireland’s fragmented health system continues to face distinctive challenges owing to the country’s complicated history of colonialism and the influence of the Catholic Church and the medical establishment on public policy. These and other factors have contributed to Ireland being one of the only European Union (EU) member states without a fully realised universal health system [1, 2]. Differential availability of health and social care services and a system overly dependent on hospital care are exacerbating inequalities in access to care. Despite ongoing reforms, the nation’s health system grapples with perennial ‘crises’ and there is an urgent need for comprehensive systems change [3].

The significance of governance in the successful operation of health systems is well-established, particularly in terms of the coordination of multiple and diverse actors [4], enhancement of policy performance and implementation [5], and the determination of decision-making pathways and resource allocation, all of which can work to either facilitate or constrain policy development and implementation. In short, as Travis and colleagues neatly put it, “the ‘quality’ of governance affects the environment within which health systems operate” [6: P3].

While good governance is increasingly recognised as crucial to the functioning of health systems [5], how it is operationalised—how governance functions in practice—and how the various aspects of governance interact with complex processes involved in the implementation of health systems *reform* specifically, remains largely overlooked in the literature [7]. Health system reform is a challenging and complex undertaking and is inextricably linked to effective governance [8, 9]. To better understand the role of governance in major health system reform efforts, the research presented in this paper examines the case study of the establishment of new health regions in Ireland between 2018 and 2023. Through document analysis and interviews with key informants, it investigates how aspects of governance have worked to shape the design and implementation of the new regional health organisations.

## Conceptualising governance in health systems analysis

Since the 1980s, the study of governance in both public and private sectors has gained prominence in the academic literature [10, 11]. Expanding application of the concept has led to diverse terminologies and models, resulting in a degree of conceptual ambiguity [12, 13]. Distinct forms of governance (e.g. market, hierarchy and network) have their origins in various fields, such as economics, political science, and sociology [14–16]. Additionally, the scope of analysis ranges from examining governance arrangements of context specific individual projects to that of supra-national, multi-level governance structures such as the EU [17]. This research draws on Greer and colleagues’ work on health system governance, adopting their definition of governance as “the process and institutions through which decisions are made and authority in a country is exercised” [5]: 28). We applied Greer et al’s TAPIC framework with which to conceptualise health governance because in developing it, the authors set out explicitly to synthesise “key elements of governance that have been identified and validated in the enormous literature, and by so doing help policy-makers identify a road map that can allow for the practical analysis of governance issues” [18]: 9. We focus on the processes and institutions through which decisions were made and authority exercised related to the development of new regional health organisations in Ireland in the years 2018 to 2023, a period during which the country’s health system experienced multiple challenges—not least the major disruption caused by the COVID-19 pandemic.

The study’s data collection and analysis are anchored in the TAPIC framework of healthcare governance [5, 18]. TAPIC appraises governance across five pivotal domains: Transparency, Accountability, Participation, Integrity and (Policy) Capacity [5, 18–20]. These five dimensions are treated as facets or parts of governance rather than as idealised targets, emphasising that while each have positive connotations, more is not necessarily better [5, 18]. Having laid out the conceptual grounding for the research, a short overview of Ireland’s healthcare system, the specific context for our study, follows.

## The evolution and challenges of Ireland’s healthcare landscape

### Towards a universal healthcare system

Ireland’s healthcare system has undergone repeated reform efforts in recent decades to address the needs of a rapidly growing and ageing population and keep abreast with clinical innovations and advancements in care service delivery [21]. Yet Ireland remains an anomaly as the only EU member country without universal primary care and one of the few still to realise universal health coverage (UHC), despite government policy intent since at least 2011 [22, 23]. Its fragmented, two-tier healthcare model creates a divide between those who can afford to pay out of pocket for private health insurance coverage and faster private care and those who cannot [22].

The pressing need for healthcare reform in Ireland has been voiced both within and outside government, particularly in recent years as the Irish health system has experienced significant capacity, access, and quality crises [21, 24–27]. Reaching a consensus on how best to implement health and social care reform to achieve UHC remains a challenge, however. UHC is defined by the World Health Organization as ensuring “that all individuals and communities receive the health services they need without suffering financial hardship. It covers the full spectrum of essential, good quality health services, from health promotion to prevention, treatment, rehabilitation and palliative care” and is firmly rooted in the notion of health as a human right [28]. While multiple prior attempts to introduce universal healthcare in Ireland in one form or another were unsuccessful [21, 29], a breakthrough came in 2017 with an all-party political consensus blueprint detailing a path towards a universal healthcare system for Ireland, referred to as the Sláintecare Report [30]. Based on the recommendations outlined in the Oireachtas blueprint or report, in 2018 government adopted *Sláintecare*, Ireland’s ten-year reform plan (‘Sláinte’ translating to ‘health’ in Irish) [30, 31].

At the time of writing, over five years into the implementation of Sláintecare, progress has been inconsistent and slow [22, 32, 33]. However, the post-COVID-19 era seems to have injected renewed momentum into aspects of the reform programme and certain key actions have been progressed, including the abolition of in-patient hospital charges, expansion of eligibility for free GP services, and a new public-only contract for medical consultants [27, 34, 35].

Central to Sláintecare’s vision is a decentralised health service wherein resources are allocated based on population health need, and delivery is integrated across primary, acute and social care services and settings [36, 37]. The introduction of new regional health structures, known as Health Service Executive (HSE) Health Regions (previously termed Regional Health Areas, RHAs), planned for the first months of 2024, represents a significant structural reorganisation of the health service. It is proposed that the six new HSE Health Regions (‘Health Regions’ going forward) will be responsible for planning, dispersing allocated funding, managing, and delivering healthcare within their respective borders [38].

## Methods

The study employed a qualitative multi-method approach, combining document analysis and key informant interviews to explore the governance mechanisms underpinning the development of Health Regions in Ireland. The research presented in this paper forms one workstream within the Health Research Board-funded *Foundations for Sláintecare* research project [39, 40]. While the project’s initial aim was to coproduce research informing the design of new health regions, with the arrival of Covid-19 and the suspension of the regions’ reform, the project aims pivoted to harness key learnings from Ireland’s health system response to COVID-19 [21, 34–36, 39, 41–43]. Ethical approval was granted by the Research Ethics Committee of the Centre for Health Policy and Management and Centre for Global Health at Trinity College Dublin [3].

### Document analysis

A document analysis was conducted entailing the systematic examination of policy documents, government reports, white papers, and official statements related to the establishment and governance of the new Health Regions between 2018 and 2023 as well as any relevant academic literature [44]. Data were collated and analysed to identify governance structures and processes and to illuminate implicit, unanswered questions regarding the design, operation, and oversight of the Health Regions. A list of key policy documents related to Sláintecare and the Regions is included as a table in a supplemental file [see Additional File 1]. The document analysis was initiated at the start of the study and simultaneous data collection and analysis continued until data collection was drawn to a close in fall 2023. The document analysis portion of the study informed the development of the interview guide for subsequent interviews with key informants, identifying key areas where further insights were sought.

### Key informant interviews

**Fig. 1:**
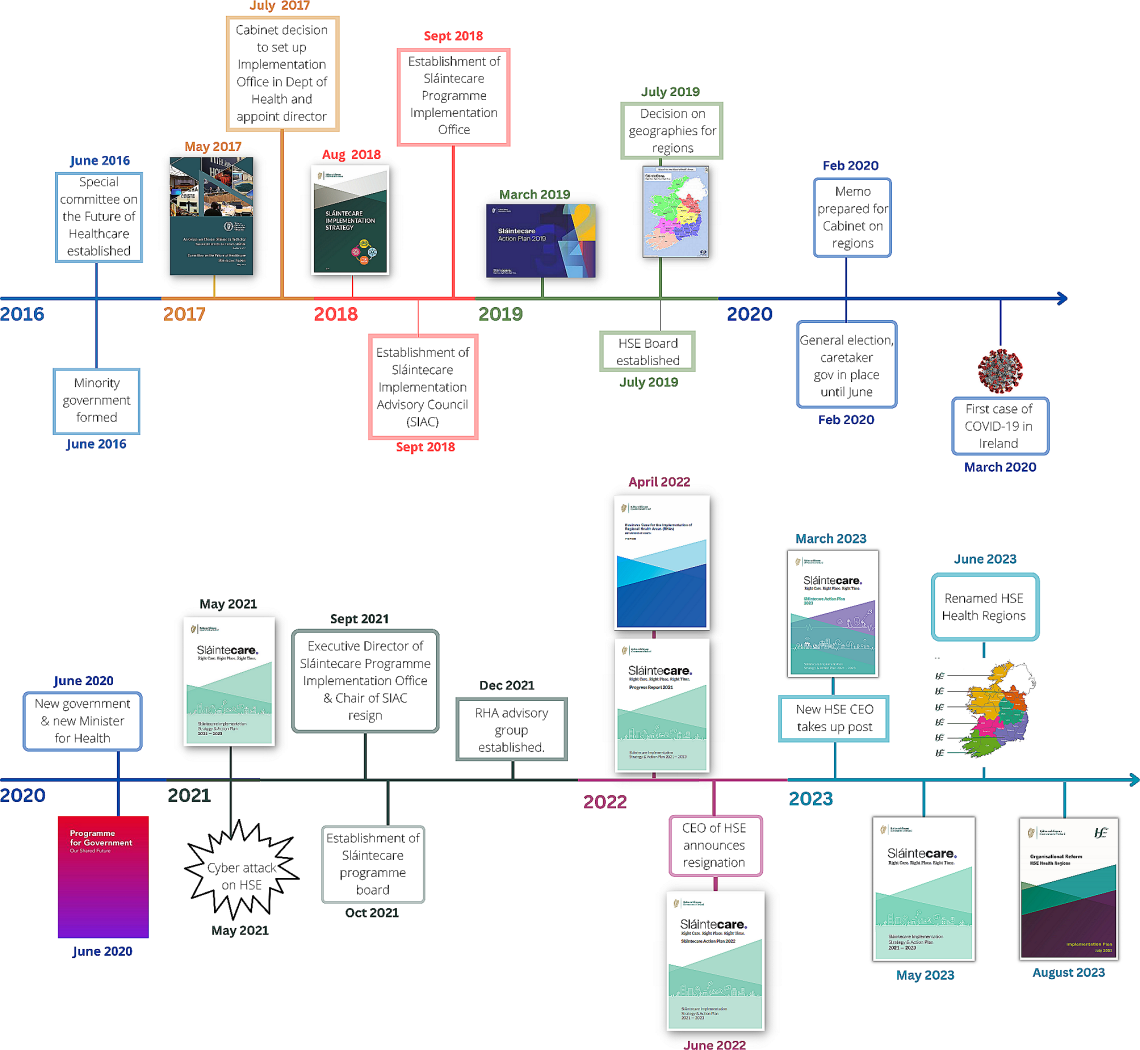
Timeline of policy developments related to HSE Health regions

To delve into the ‘black box’ of governance processes, 12 semi-structured key informant interviews were conducted between November 2022 and August 2023 [45]. This period coincided with a number of significant developments in the reform process, including the arrival of new leadership at the HSE and publication of the Implementation Plan for the re-named Health Regions (see Timeline presented in Fig. 1). The research team was thus able to garner interview participants’ ‘real-time’ reflections on ongoing developments related to the reform. The selection of interviewees was purposive [45, 46], targeting individuals with direct involvement or informed perspectives on the governance of the Health Regions, including an equal number of senior policy makers and health system leaders (see Table 1). Given the small number of people involved in this reform process, and to ensure confidentiality is maintained, the information provided on the profile of key informants is purposefully limited. Interviews sought to uncover the experiences, insights, and perceptions of these expert stakeholders, focusing on the practicalities of governance and areas that remain under-examined or contested. As a number of the key informants who participated in the study were still actively involved in the reform process at the time of the interview, the interviews provided an opportunity for them to reflect on the reform process, creating a feedback loop between research and policy-making. Given the embeddedness of the research in the reform process, the researcher conducting the interviews made a concerted effort to avoid biasing interview participants and by extension the research process by asking non-leading and open-ended questions and by refraining from expressing their own views. Each interview was recorded with consent, transcribed verbatim, and pseudo-anonymised to maintain confidentiality.

The research team used data triangulation to limit the introduction of both key informant interviewees and researcher bias into the data collection and analysis. Where possible and available, the research team asked key informants for documentary evidence to support claims made during the interview. In addition, the research team aimed for a diversity of perspectives in its purposive sampling approach, recruiting participants outside the ‘inner circle’ of the reform policymaking process.

**Table 1:** Key informant interviews by personnel type

Interview No.	Personnel type
1.	Health official/policy maker
2.	Health official/policy maker
3.	Health system leader/manager
4.	Health official/policy maker
5.	Health system leader/manager
6.	Health official/policy maker
7.	Health system leader/manager
8.	Health official/policy maker
9.	Health system leader/manager
10.	Health official/policy maker
11.	Health system leader/manager
12.	Health system leader/manager

### Analytic framework and approach

Data from both the documentary analysis and key informant interviews were coded using a primarily deductive approach in order to capture how the different aspects of governance defined in the TAPIC framework were implicated in the data. The research team created a predefined analytical matrix corresponding to the TAPIC framework [5, 18], encompassing Transparency, Accountability, Participation, Integrity and Capacity as analytic categories (see Table 2). This framework facilitated a structured and focused analysis, allowing for the categorisation of data into these five dimensions of governance. Coding was performed iteratively, with initial codes refined and sub-codes developed as patterns were identified, in keeping with thematic analysis methodology [46]. While taking a primarily deductive approach, the research team also used inductive coding, facilitating the identification of additional concepts and phenomena in the data that transcended the predefined analytic categories. Microsoft Excel was utilised to support the coding process, ensure methodological rigour, and enhance the reliability of the findings.

**Table 2:** How the TAPIC Framework was Operationalised for Analysis

TAPIC Dimension	Conceptualisation
**Transparency**	Pertains to the manner governing entities communicate decisions to the public and other external actors. Instruments fostering transparency include committees, routine reports, and performance evaluations.
**Accountability**	Encompasses justification and redress. It emphasises the nexus between an actor and a forum (e.g. a legislature) where decisions are reported, explicated and possibly sanctioned. Mechanisms buttressing accountability encompass contracts, regulations and ethical guidelines.
**Participation**	Refers to the processes through which affected parties are integrated into decision-making processes, thereby having a genuine stake in the operations of governing institutions. Vehicles for participation include stakeholder meetings, public consultations, and advisory panels.
**Integrity**	Signifies clarity in role allocation, adherence to formal rules and stability, epitomising a proficient bureaucracy. Instruments enhancing integrity encompass internal audits, precise personnel policies and stringent administrative protocols.
**(Policy) Capacity**	Primarily addresses policy capacity, aligning policy development with available resources to achieve set objectives. This domain covers the technical apparatus available to top-tier policymakers. Capacity augmentation tools include performance analytics, incorporation of expert advice and staff technical training.

Table content adapted from Greer et al., 2016; 2019.

The triangulation of document analysis and key informant interviews, coupled with the systematic application of the TAPIC framework, yielded rich findings as to the governance dynamics at play in the reform of Ireland’s health system. These findings, detailed in the following section, provide empirically grounded insights for health system policy-makers, professionals, and leaders.

## Findings

Analysis of public documents and interviews with key informants reveal a reform process that has experienced many challenges. As the findings below illustrate, these challenges were brought on by issues directly related to the governance arrangements of the reform, as well as by circumstances indirectly related to governance, notably the COVID-19 pandemic and the strain it placed on already limited government and health system resources—both staff time and financial resources—as well as a cyber-attack targeting the HSE in May 2021. The findings go on to suggest an emerging period of institutional stability, albeit one in which clear governance challenges and divisions remain.

### Analysis of the health region reform policy process

Following publication of the Sláintecare Report in 2017 [30], the Sláintecare Implementation Strategy was published in 2018 [31]. The Sláintecare Implementation Advisory Council (SIAC) and the Sláintecare Programme Implementation Office, helmed by an Executive Director appointed by the Minister of Health, were established to progress the implementation [47]. In a consequential early decision by government, the Sláintecare Implementation Programme Office was set up within the Department of Health rather than in the Department of An Taoiseach (Prime Minister) as originally proposed in the 2017 Sláintecare Report [30].

The following year saw the release of the first Sláintecare Action Plan and the geographical delineation of the new Health Regions in July [48, 49]. The stated rationale for the Health Regions was that devolving certain aspects of decision-making authority to the regional level and closer to the frontline of care provision, would enable achievement of the broader objectives of the Sláintecare reforms [49]. These objectives include enabling more streamlined and improved clinical governance, achieving better integration of care services and care pathways, improving corporate governance and accountability, and the development of a population-based approach to service planning and funding in place of the existing care groups funding model [49]. While institutional structures and objectives for implementation were in place at this stage, and stakeholder engagement within the system had been initiated, all progress and activities related to the regions was brought to an abrupt halt with the onset of the COVID-19 pandemic in March 2020 [3].

Amidst pandemic challenges, May 2021 marked the launch of a new Sláintecare Implementation Strategy and Action Plan [37]. The plan cited the development of regional health areas as one of four discrete ‘projects’ but included scant detail on how they might be operationalised [37]. Notably, the plan’s publication was closely followed by the resignations of the Executive Director of the Sláintecare Programme Implementation Office and the Chair of the Sláintecare Implementation Advisory Council [50]. Both cited concerns over reform progress and internal conflicts over the extent of institutional and political support for the reforms as well as the slow timeframe. The Executive Director’s letter of resignation, leaked to the media named the regional health areas as one of three key areas requiring dedicated, focused reform effort [50]. The letter stated, “these reforms require a governance and oversight structure other than that which exists at present” [50].

In the wake of the resignations, the Health Minister initiated substantive changes to the reform programme’s governance and decision-making arrangements, dissolving the Implementation Office and SIAC and introducing a new Sláintecare Programme Board to be co-chaired by the HSE CEO and the Secretary General of the Department of Health [51]. This was followed by the establishment of the Regional Health Areas (RHA) Advisory Group later in 2021, chaired by the CEO of the Medical Council of Ireland [52].

In 2022, the Department of Health’s Business Case for the regions recommended that the Health Regions—then still referred to as Regional Health Areas (RHAs)—should be administrative divisions within the HSE structure rather than separate legal entities [53]. The Sláintecare 2022 Action Plan, released in May 2022, aimed to finalise the RHA implementation by year-end with consultations planned for late 2022, to be led by a management consultant firm [54]. During this period the HSE CEO announced his resignation citing personal reasons, bookending a series of high-profile exits and wider changes at senior levels within the public health system. A new HSE CEO was appointed in March 2023. As work on the regions progressed, RHAs were renamed Interim HSE Areas, only to be renamed again with the publication of the long-awaited implementation plan for the regions in August 2023, this time to HSE Health Regions [38].

The August 2023 implementation plan determined that unlike the Health Boards regional structure that existed in Ireland between 1971 and 2005, the Health Regions would not be granted distinct legal status (i.e. will not have their own boards) [38]. Instead, the HSE would undergo an internal reorganisation into six regions, with each region led by a Regional Executive Officer (REO) reporting directly to the CEO of the HSE and the HSE Board [38]. The planned reform thus involves a form of administrative decentralisation or de-concentration of some of HSE Centre functions to the level of Health Regions [see for example 55, 56].

The policy developments analysed above and presented in the timeline below (see Fig. 1) suggest a lack of political commitment prior to and during COVID-19, as well as conflicts at the Centre (i.e. the central government institutions involved - DOH centre, HSE centre, Department of Public Expenditure and Reform (DPER), Department of An Taoiseach and the cabinet of government ministers - from here, the Centre). This contributed to senior resignations, competing policy visions, and frequently changing institutional and decision-making arrangements that had a destabilising effect. The findings presented in the following section are situated within this policy context and detail the complex interplay between health system governance and health system reform as it relates to the TAPIC governance domains.

### Analysis of the governance of the regionalisation reform

#### Transparency

Public access to policy documents is a critical component of transparent and accountable governance [57]. Despite reporting mechanisms being in place that have the potential to enhance transparency around key decisions and processes, several concerns of transparency emerged from the data. It was often not clear to interviewees, all of whom hold senior-level positions across the health system, where decision-making regarding the design and implementation of the HSE Regions stood, and what the grounds or justifications for said decisions had been, leading to a generalised sense of confusion. This was especially the case for those Interviewees operating outside the Centre. One interviewee, a senior system manager, expressed frustration with what they perceived to be a lack of information sharing on the part of the Centre, particularly in light of the short timeframe for establishing the new Health Regions:

“… it took maybe the full year, [before] we got a draft… of [the] implementation [plan], it wasn’t an implementation plan […] it’s a real concern in that… we’ve been told is that 2024, standing up in 2024 will be a transition year.” [I09].

Relatedly, transparency in the form of communication among the various stakeholders involved in the reform was generally perceived to have been poor in the period under consideration. This was true for decisions taken by the Health Department, not properly communicated to partners in the HSE, and perhaps more significantly, to stakeholders outside the Centre, including clinical managers and frontline staff, again leading to widespread confusion and uncertainty, and a lack of awareness of the latest developments in the design and implementation of the new Regions. Responding to the interviewer’s question on the extent to which those responsible for the design and implementation are keeping key stakeholders informed of decisions, another senior health system manager suggested:

“I think they’re certainly trying to [keep stakeholders outside the Centre informed] …I would hazard a guess that one of the reasons is that decisions haven’t been made. So therefore there’s nothing to communicate…people kept saying, there’s a plan in a drawer somewhere, somebody has this done, and they’re only going through all this engagement…just to tick a box, and I kept going, no, they haven’t…from my perspective, that was actually far more worrying.” [I11].

The lack of an integrated digital platform for the health system, and lack of access to basic technologies such as computers/laptops and dedicated email for many HSE staff were cited as a significant obstacle to communication between the Centre and frontline staff and thus to transparency, stating:

“…half of the [HSE frontline] staff literally don’t have access to computers. So it is difficult to get communication right out into the wider health environment. Other than through public media, we often take criticism for saying or distributing information centrally without it being known locally…So we do have to think hard about how we get information actually down to the ground” [I07].

Reporting and monitoring requirements were said by many interviewees to be in development for the implementation and future operationalisation of the regions. Yet the choice of progress indicators and the extent to which these have been or will be effective in holding individuals and organisations accountable was questioned by some interviewees, for example:

“I don’t think we’re outcomes-focused enough…I think we place too much emphasis on [hospital] trolleys. And we don’t place enough value on health promotion, prevention, improvement, and what health value are we adding?” [I11].

Furthermore, in several cases, key documents pertaining to the design and implementation of the Regions did not appear to be available in the public domain. When asked, interviewees were often unsure if a long-promised implementation plan for the Regions had been published in the public domain or not, which contributed to the sense that decisions were not being taken systematically or transparently. Even if a document was in the public domain, it was not always easily accessible or visible, a point supported by the efforts of the research team to track down specific relevant documents for the analysis.

Finally, the documentary analysis revealed that implementation progress measures and indicators changed across annual action plans and progress reports, without acknowledgement or explanation [54, 58]. Appropriate, effective and consistent reporting and monitoring systems are essential to ensuring transparency of governance processes, and in turn for accountability of same [59].

### Accountability

Accountability is a core, yet complex, function of governance [60]. The documentary and interview analysis reveal a complicated, evolving picture of the accountability structures and mechanisms in place. Early political decisions led to the establishment of the Sláintecare Implementation Programme Office in the Dept of Health not the Department of An Taoiseach as originally envisaged in the 2017 Oireachtas report. It also saw the establishment of a HSE Board in 2019 [61]. While it seemed clear to most interviewees that responsibility and accountability for the design of the Regions rests with the Department of Health, and ultimately with the Secretary General reporting to the Minister for Health, there does not appear to be a system or a culture of reward or sanction, of *holding* senior leadership *accountable* for decisions taken and implementation.

Accountability for the implementation, for realising change and operationalising policy, was much less clear and more contentious, as evidenced by repeat governance changes during the period of this research and as described above. Responding to the question of whether it was clear who is ultimately accountable for the decentralisation reform, one senior official said:

“[…] some of the problems of the Regions is that there is a sense of this is about reorganising the HSE […] And we’ve repeatedly [said] that this is not about the reform of HSE. It is reform of the health system. And there is a multiplicity of organisations way beyond the HSE. So if this was the HSE’s to implement, and you can say, “okay, guys go off and do that” you’d have the problems of them, trying to hold on, in the Centre, as much power as they possibly could […] if you think about this, and the regionalisation as a reform of the entire system, then it’s not as clear … who is responsible for that? Because it’s a complex adaptive system … the governance issue becomes much more critical. So that, to me, that’s a very simplistic thing, the SecGen [Secretary General of the Department of Health] and the CEO of health service [HSE], clearly there’s very significant roles to be paid by both but it’s not sufficient.” [I04].

Furthermore, the accountability structure outlined in the 2022 implementation plan for the Health Regions is not clear and has the potential to cause problems down the line in the establishment and operation of the new regions [38]. This view was supported by multiple interviewees who pointed out a possible future source of tension between REOs and the HSE CEO and HSE Board when it comes to budgetary autonomy of the Regions. As the new Health Regions will not have separate legal status, the HSE CEO and HSE Board will have ultimate accountability and authority, and it remains to be seen how regional leadership will react if they perceive an incursion on their autonomy. One Interviewee raised the critical point that in the absence of separate legal status for the Health Regions, there would need to be clear accountability mechanisms in place between the REOs and the HSE CEO:

“…as long as [the new HSE CEO] is … the CEO [of the HSE], [he] is legally responsible for everything in the HSE … the design has to ask the question, how does the CEO get assured that things are okay? Or have sufficient knowledge to plan and performance manage that environment?” (I07).

Crucially, a number of interviewees raised the (lack of) accountability of the Department for Public Expenditure and Reform (DPER) in the decentralisation process. Despite having outsized influence on the allocation of resources both for the implementation and the future operation of the six Health Regions, according to one interviewee, DPER behaves almost as a silent partner, with little understanding of health service operations and clinical governance, of how health systems operate on a day-to-day basis and the inherent complexity of people’s health and social care needs. A senior health system manager articulated DPER’s significant influence on and simultaneous lack of accountability for the Health Regions’ design and implementation:

[ “…DPER take an awful lot of decisions that impact hugely on the services, but yet they don’t carry any risk with it, they can all go to bed at night and sleep fine and all the rest of us are up worrying going dear god, what’s going to happen with this?” [I11].

### Participation

With one or two exceptions, interviewees felt that efforts to engage relevant stakeholders had been lacklustre to date (the last interview took place in August 2023), stating that stakeholder participation had been widely treated as a box-ticking exercise as opposed to considered or ‘real’ engagement. Several Interviewees stated that COVID-19 played a significant role in halting positive stakeholder engagement initiatives led by the Sláintecare Implementation Office prior to the onset of the pandemic in late 2019 and early 2020, a point supported by the documentary analysis. One senior health official reflected:

“I wish that I would have started talking to regions, like, talking to CHO and hospital folks way earlier in the process…” [I02].

Knowing when to engage relevant stakeholders and which stakeholders to engage at the various stages of the design of the regions and the design of the implementation was cited as an important aspect of meaningful participation by several Interviewees. When done well, Interviewees said that it can provide critical feedback and input to the design phase. In response to the interviewer’s question about how participatory the process had been pre-pandemic, one Interviewee, another senior health official, countered with the caveat that participation must be appropriately timed:

“At that point, no [the process was not participatory], but I don’t think it needed to be at that point…it was very abstract, and it’s very hard to go out and engage with people in the abstract…I know early engagement is important. But we just weren’t at that point.” [I01].

Interviewees also stated that meaningful participation is vital to ensure buy-in and ownership of the policy. While the TAPIC framework [5, 18] refers to members of the public, and to people ‘directly affected’ by the end-users in its Participation domain, multiple interviewees referred to the need to bring relevant people to the table from *within* the civil service itself, across different departments/units, as well as from the wider health system. In a clear link with the (Policy) Capacity and Accountability domains, interviewees questioned the role of management consultants being contracted to carry out stakeholder engagement activities related to the Health Regions’ design and implementation. It was suggested that they were contracted because the Centre did not have the in-house resources or capacity to carry out crucial stakeholder engagement activities themselves. One interviewee noted that it is preferable to have people undertaking such activities from within the system, people who ‘know the system’:

“…there was a huge infrastructure transformation needed and they’re gonna have to bring in people right, but it has to be led and driven by people within the system. And people need to see that. They can be supported and enabled … they don’t have to do all of the background work… But there is no way that it should be… here’s a group of consultants that can do this”. [I04]

### Integrity

Without exception, Interviewees stated explicitly or implied (by expressing confusion) that clarity around the aims of the decentralisation reform and the policy vision for the Health Regions was lacking. This contributed to blurring roles, responsibilities and processes, in particular the division of responsibilities between the Department of Health and the HSE for implementation of the reform itself and for governance of the future Health Regions, including crucially, clinical governance. As a result, interviewees expressed a lack of faith or trust that the Health Regions would be implemented successfully and in a timely manner by senior leadership, and that it would fulfil the ambitious if rather vague goals ascribed to it. One senior health system manager said:

“[…] there’s this talk about… devolve decision making, accountability, autonomy and responsibility to the regions. Lean centre … I think the HSE Centre has yet to be clearly defined. And I think that’s a problem because if you have people in the Centre still trying to manage the regions then the regions aren’t being given that autonomy and responsibility and accountability, that they’re signing up for.” [I09].

Another senior health official spoke to how disagreements on the vision for the Health Regions have undermined the effective governance of its design, implementation, and potentially, of its future operation:

“… the first thing was that all of these different parties involved in [the regions] had a completely different understanding and perspective on what this was - so there was deep resistance. So when you have that, whatever governance structure you have in place, won’t resolve it […].” [I04].

Interviewees also questioned the very rationale of the reform. While stating that they were in favour of devolving authority to regional health structures in principle, some of those interviewed expressed uncertainty about whether the international evidence was strong enough to justify the enormity of the undertaking. They expressed uncertainty as to the precise rationale for devolvement and expressed the view that decentralisation alone would not bring about integrated care, nor better outcomes for service users.

“I still don’t think that we’re clear on what, we would … like… in five years’ time, having invested all this time and effort in going to the regions, what are the two things that are going to be different? That’s not visible to me. … we all go oh devolved decision making, … close to patients, population-based planning … but what does all that mean? …it’s big and complex, getting clear on purpose and benefits that’s … the next big lesson.” [I05].

Interviewees alluded to a tension between those working at the Centre who spoke about the reform as ‘simply’ a reorganisation or restructuring of the HSE—a view especially prevalent among the senior leadership of the Department of Health—and those who viewed the reform as requiring broad systems level change. One senior health official stated:

“Post-COVID, we have tried to make it as co-designed a process between the HSE and the Department as possible…there’s a significant recognition this is a reform of our system, of which we all have standing and have a part, and there’s a recognition that the Department’s relative role and responsibility will have to change as…they are devolving particular functions that they currently hold on to entities, namely the regions.” (I02).

And another senior official expressed a similar view, saying:

“[…] we had the leadership in the HSE that absolutely did not believe in regionalisation in any kind of meaningful way. […] and you had a SecGen who was thinking yeah regionalisation, but with minimal disruption. […] even if you had whatever governance you’d put in place, then it would meet up against these very significant blockages, both overt and covert. […] the number one thing was about leadership, if you don’t have people in to understand it as a complex system in the first place. So putting somebody in charge, and saying so you are going to do this. They cannot do that if you’ve got very significant power brokers in different parts of the system.” [I04].

It should be noted that the interviews took place over a ten-month period and the status and structure of the Health Regions was finalised and made public towards the end of this period with the publication of the regional implementation plan in August 2023 [38]. There was significant confusion on the matter among Interviewees, until publication of the plan. While the decision not to grant the Health Regions separate legal status, to have them instead reporting directly to the HSE CEO and Board was only confirmed in August 2023, this is a position that had been favoured by the Department of Health since at least April 2022 with the publication of its Business Case for the new regional structures [53]. This decision indicated a preference on the part of the Department of Health and government to characterise the decentralisation reform as a contained organisational restructuring of the HSE, rather than a system wide reform. Some interviewees indicated that the DOH decided to retain accountability for health and social care service delivery with the HSE Centre in order to avoid the risk of regional variation in clinical standards and retain a degree of centralised clinical oversight.

### (Policy) capacity

Within its Capacity domain, the TAPIC framework focuses on *policy* capacity [5]. According to Greer et al. [18], policy capacity refers to “the ability to do the staff work and analysis to turn a political idea into a thought-out proposal, or explain why it is risky” (10). The emphasis is very clearly on expertise in policy-making or in the drafting of policy, and not on reform or change capacity and expertise. The data suggest that while there was adequate policy formulation capacity in the system, *implementation* capacity, the know-how to translate policy into practice, was/is inadequate. Responding to the interviewer’s question about whether there is sufficient policy capacity in the system, a senior health system manager responded that while policy capacity has improved, it is the expertise to know what to do with the evidence, for example, or how to operationalise evidence that is currently inadequate:

“… they’re much better [at policy] now than they would have been three or five years ago. Are they at the end point? Absolutely not. But I do think they have…more information now than we ever did. And really… [the question] is … are we using it correctly?” (I11).

Another senior health system manager referred to the lack of so-called implementation capacity in the system as a lack of expertise in ‘change management’:

“My own experience…is that… departments are good at what they’re there to do, which is advise the Minister, develop policy, give options and recommendations to government. What they don’t do is information and change management.” [I03].

Related to the above and a seeming lack of capacity in how to *design, plan and implement a complex programme of reform*, the data suggest that the programme of reform has distinct phases beyond ‘design’ and ‘implementation’ that have not been clearly delineated and distinguished from one another, leading to confusion and conflict as to who or which organisation is responsible for which stage.

While several interviewees noted that the Department of Health is responsible for policy and design of the Health Regions, and the HSE for implementation, this is an oversimplification. The Department of Health together with government has responsibility for determining policy, that much was clear to everyone. When it comes to other phases of the reform process, namely the design, planning how the implementation will unfold, and then operationalising that plan, however, the division of responsibilities and accountability at the Centre are highly contentious. This finding highlights the close linkages between the (Policy) Capacity domain and the Integrity and Accountability domains of the TAPIC framework.

Several interviewees pointed out the need for all staff involved in the reform, those working in the Centre as well as those external to it, to have sufficient time as a crucial - if often overlooked - aspect of capacity. Interviewees felt that such time needs to be distinct from ‘normal’ work/responsibilities to allow staff to focus on the design/implementation of the regions. This is especially challenging among non-Centre staff given the ongoing health care professionals/workforce shortage in the system. A health system manager with operational experience stated:

“I would remain concerned about the scale of resource available to truly implement effectively locally, I know well, the pressures that will come on the operating system to deliver the operational reality, so there will have to be dedicated resource to primarily focus on this.” [I07].

The importance of leadership and management capacity for governance, the capacity to manage relationships and establish trust, is another key finding emerging from the data. Several interviewees alluded to the tangible impact—in terms of progress and in terms of the quality of collaboration between the HSE Centre and the Department—that the arrival of a new HSE CEO in March 2023 had on the reform process. According to interviewees working within and external to the HSE, the CEO’s commitment to the implementation of Health Regions, and his bringing different stakeholders from within the HSE and from across the health system to the table seems to have, initially at least, alleviated some of the sharpest conflicts previously reported at the Centre. For example, from the beginning of his tenure, the new CEO brought Community Health Organisation and Hospital Group CEOs onto the newly formed HSE Senior Leadership Team in preparation for the introduction of the new regional structures. Yet in terms of formal accountability mechanisms and governance structures, his appointment alone has not changed a great deal. A senior health official stated:

“Within the HSE there was a … Battle Royale going on between different factions, a complete breakdown at the top-level, in the senior team, because it was just diametrically opposing views of what this meant and what it should be [the design of the Regions]. You can’t plan anything on that basis. […] the most critical thing was leadership because it has a huge role to play. And I think [the new HSE CEO] is definitely in … the right space. There’s other issues, but he is definitely now saying ‘here’s where we’re going.’ [I04].

## Discussion

What does the above analysis of interview and documentary data about the regionalisation reform process in Ireland in the period 2018–2023 contribute to the scholarship on the significance of governance as a concept and the role it plays in (large-scale) health system reform? The findings elucidate a number of distinct ‘lessons’ for policymakers in terms of areas to address across the five TAPIC domains of governance. We argue, however, that by engaging across domains and with the idea of health systems as complex, dynamic networks influenced by specific relational contexts [35, 62], the lessons emerging from the data for the governance of health systems and health systems reform are of even greater value. We expand on this through four key points below.

Firstly, the findings highlight deficiencies in the governance of the Health Regions policy process across all five TAPIC domains, reinforcing a range of health system governance issues identified previously in other research on the Irish context [63–65]. Issues pertaining to the Accountability and Transparency domains are especially prominent, reflecting the conflictual relationship and accountability deficiencies that developed between the DOH and the HSE following the establishment of the HSE nearly two decades ago [29, 65], and which set the stage for the ‘conflict at the Centre’ discussed in the findings. Adding to this charged dynamic is the role of DPER as a key third actor, an institution wholly outside the health governance structure, yet one with substantial power over health policy decisions. DPER has *de facto* decision-making power without any formal accountability to the health system [22], a problematic arrangement that made news following the announcement of the 2024 Budget and the stark discrepancy between funding requested by the DOH/HSE and that allocated by DPER [66].

In turn, weak accountability mechanisms and poor transparency have concrete implications for the Integrity domain, resulting in vague divisions between responsibilities for policy design versus implementation and operation. Barry and colleagues [21] highlighted similar issues in their analysis of integrated care governance, noting that competing visions had led to a lack of clarity on roles, with changing personnel compounding the problem and leading to gridlock/stasis.

Secondly, the TAPIC framework is useful for thinking about and analysing the components of health system governance in a particular setting. Our analysis revealed that the richest findings and insights emerged at the borders of TAPIC’s five domains, in the reciprocal and constantly re-producing relationships between issues of Transparency, Accountability, Participation, Integrity, and (Policy) Capacity.

Prominently, our research highlights the importance of the link between policy capacity and what we have termed ‘implementation capacity’ for health system governance, an element absent from the TAPIC framework’s Capacity domain. A lack of implementation capacity in the Irish health system is contributing to a blurring of roles and responsibilities (Integrity domain) [67]. It is also contributing to weak accountability for certain aspects of the reform process (Accountability domain), and for the future operation of the Health Regions.

The lack of clarity on roles/responsibilities could be said to originate in senior leadership’s desire to hold on to control and an unwillingness to delineate roles and responsibilities, a blame avoidance strategy rooted in an institutional culture in which a lack of transparency has historically been employed to obscure lines of responsibility [68]. This strategy reflects what Clay and Schaffer [69] term an ‘escape hatch’ at the heart of separating policy-making and implementation or policy and operations processes, whereby poor implementation, rather than weaknesses in policy-making processes in the first instance, is blamed for poor policy outcomes. A failure to invest the necessary resources in capacity — crucially in *implementation* capacity— is also a clear legacy issue with direct implications for the Health Regions reform process, not least the decision to outsource critical tasks to consulting firms. Public sector reliance on the work of consulting firms has been shown to undermine good governance, as terms and contracts often include weak accountability arrangements and operate within a ‘black box’ of limited transparency [70].

Thirdly, a formulaic approach to the study of health systems governance is not helpful, something the TAPIC framework authors also caution against [18]. There are clear interactions between TAPIC’s five domains, though it is not possible to make determinations of causality between them. This is indicative of the complex processes at play in health systems governance, and the need to embrace complexity in order to understand the functioning of health systems, a key finding of earlier work by the same research group [35].

The significance of deep context for understanding the relational dynamics involved in health system and health system reform governance, and for forging the path forward cannot be underestimated. While the importance of context for policy analysis is widely recognised, contemporary analyses of healthcare reform tend to provide static descriptions of the status quo, or to provide surface level detail of major developments and milestones over the years. Further, historical context and the intricacies of institutional relationships shaping current health structures and processes are usually neglected, resulting in policy blind spots and the repetition of missteps and miscalculations, and a broken record of ‘lessons learned’. Historical analysis is vital to understanding the relationships between key institutions and key actors involved [71], including for the regionalisation reform discussed in this paper. The current structural reforms are only the most recent in a long line of centralisation/decentralisation reforms in the history of Ireland’s healthcare system, which in each instance have been undertaken to shift decision-making control and power from one set of institutional actors to another (or others) [1, 29, 72, 73].

The establishment of the HSE in 2005 was itself part of a reform aimed at *centralising* health system governance following a period beginning in the 1970s in which regional health authorities—and local politicians—were key decision-makers [29]. At the time, Tussing and Wren identified a number of issues with the establishment of the HSE and its relationship to the Department of Health, not least deficiencies in accountability arrangements between the two bodies and the scope this left for confusion and blame. These developments laid the foundation for the conflict at the Centre reported by interviewees and is evident in the contested plans and vision for the design of the Health Regions described in the documents analysed in this study.

Fourthly, this research highlights the importance of distinguishing analytically between governance of health systems in periods of relative stability, and governance of large-scale health system *reforms*. Reform is a complex process—or more accurately, a set of complex processes embedded in an already highly complex system—that requires substantial investment and specific expertise or capacity. There is a great deal to be learned from the critical implementation science literature, which foregrounds the relationality of implementation processes and the building of trust among stakeholders involved as critical to the success of any implementation programme [74]. This finding is in line with prior evidence from the Irish context that enhanced trust and communication between different system levels significantly improved universal access to integrated care during the COVID-19 pandemic [35], and is bolstered by the increasing focus at the international level on the concept of trust and its importance for large scale health system transformation [75]. In addition, analysis of the Health Regions reform process demonstrates the need for sufficient, dedicated implementation capacity. Furthermore, the analysis calls for an approach to resourcing implementation that prioritises relationships and trust building as the starting point and as the active focus of implementation teams. Further research should bring concepts of health system governance and critical implementation science into conversation with one another.

## Conclusions

Good governance is vital to the functioning of strong health systems and is critical during large scale reform. Through in-depth analysis of the evolving governance arrangements and design of the Health Regions in Ireland, this research reveals the complex interplay between health system governance and health system reform. As the findings illustrate, governance deficiencies and conflicting policy visions can engender discordance, uncertainty, and distrust at a time when they are most needed. Findings equally illustrate how leadership, institutional stability, collaboration and clarity of vision and responsibility can go some way to resolving governance issues in such domains as Integrity and Capacity, in turn advancing large scale structural reform. Taken together, this research highlights the dynamics and tensions that influence and shape health policy decisions, offering important insights not only for Ireland but also for other countries grappling with similar challenges or opportunities concerning governance of health system reform. Finally, further research on the bi-directional relationship between research and policymaking—with policymakers and other stakeholders as coproducers of knowledge and evidence, and researchers as stakeholders and contributors into the policy process—would add to the growing body of evidence that considers the practice, benefits and challenges of co-producing knowledge to inform policy and strengthen health systems.

### Electronic supplementary material

Below is the link to the electronic supplementary material.

Supplementary Material 1

Supplementary Material 2

## Data Availability

The qualitative dataset (interview data) generated and analysed during the current study is not publicly available due to the small number of people involved in the policy process under analysis and the sensitivity of the information provided by interviewees. The data are available from the corresponding author on reasonable request.

## References

[1] Barrington R (1987). Health, medicine & politics in Ireland, 1900–1970.

[2] Malone P, Millar M (2020). The only equality is the pain: an exploration of the Irish policy sphere’s approach to access and entitlement in health care. Social Policy Adm.

[3] Burke S, Thomas S, Stach M, Kavanagh P, Magahy L, Johnston B, Barry S. Health system foundations for Sláintecare implementation in 2020 and beyond ? Co-producing a Sláintecare living implementation Framework with evaluation: learning from the Irish health system?s response to COVID-19. A mixed-methods study protocol [version 1; peer review: 2 approved]. HRB Open Res 2020, 3. 10.12688/hrbopenres.13150.1 PMC7934093 33728398

[4] Moulton S, Sandfort JR (2017). The Strategic Action Field Framework for policy implementation research. Policy Stud J.

[5] Greer SL, Wismar M, Figueras J, McKee C. Governance: A Framework. In *Strengthening Health System Governance: Better Policies, Stronger Performance* Edited by Greer SL, Wismar M, Figueras J. Berkshire, England: Open University Press; 2016.

[6] Travis P, Egger D, Davies P, Mechbal A (2002). Towards better stewardship: concepts and critical issues.

[7] Buse K, Mays N, Walt G (2012). Making Health Policy.

[8] Khan S, Vandermorris A, Shepherd J, Begun JW, Lanham HJ, Uhl-Bien M, Berta W (2018). Embracing uncertainty, managing complexity: applying complexity thinking principles to transformation efforts in healthcare systems. BMC Health Serv Res.

[9] World Health Organisation (2007). Everybody’s business -- strengthening health systems to improve health outcomes: WHO’s framework for action.

[10] Levi-Faur D. From big government to big governance. In *The Oxford Handbook of Governance* Edited by Levi-Faur D. Oxford, UK: Oxford University Press; 2012: 3–18.

[11] Cheffins BR. The History of Corporate Governance. In *The Oxford Handbook of Corporate Governance* Edited by Wright M, Siegel DS, Keasey K, Filatotchev I. Oxford, UK: Oxford University Press; 2013: 46–64.

[12] Barbazza E, Tello JE (2014). A review of health governance: definitions, dimensions and tools to govern. Health Policy.

[13] Lee K, Kamradt-Scott A (2014). The multiple meanings of global health governance: a call for conceptual clarity. Globalization Health.

[14] Williamson OE (1975). Markets and hierarchies: analysis and Antitrust implications: a study in the Economics of Internal Organization.

[15] Rhodes RAW (1997). Understanding governance: policy networks, Governance, Reflexity and Accountabilty.

[16] Smith PC, Anell A, Busse R, Crivelli L, Healy J, Lindahl AK, Westert G, Kene T (2012). Leadership and governance in seven developed health systems. Health Policy.

[17] Bache I, Flinders M. Themes and Issues in Multi-level Governance. In *Multi-level Governance* Edited by Bache I, Flinders M. Oxford, UK: Oxford University Press; 2004: 1–12.

[18] Greer SL, Vasev N, Jarman H, Wismar M, Figueras J. European Observatory Policy Briefs. In *It’s the governance, stupid! TAPIC: a governance framework to strengthen decision making and implementation* Copenhagen (Denmark): European Observatory on Health Systems and Policies © World Health Organization 2019 (acting as the host organization for, and secretariat of, the European Observatory on Health Systems and Policies). 2019. 32045179

[19] Greer SL, Vasev N, Wismar M (2017). Fences and ambulances: Governance for intersectoral action on health. Health Policy.

[20] Greer SL. Organization and Governance: Stewardship and Governance in Health Systems. In *Health Care Systems and Policies* Edited by Ginneken Ev, Busse R. New York: Springer; 2018: 1–9.

[21] Barry S, Stach M, Thomas S, Burke S. Understanding service reorganisation in the Irish health & social care system from 1998 to 2020: lessons for reform and transformation [version 1; peer review: 1 approved with reservations]. HRB Open Res 2021, 4.

[22] Connolly S, Wren M-A, Keegan C. Towards universal healthcare in Ireland – what can we learn from the literature? ESRI; 2023.

[23] OECD/European Observatory on Health Systems Policies: Ireland: Country Health Profile. 2021. https://www.oecd-ilibrary.org/social-issues-migration-health/ireland-country-health-profile-2021_4f7fb3b8-en; 2021.

[24] Department of Health and Children (2001). Quality and Fairness. A Health System for you.

[25] Department of Health and Children (2001). Your views about Health. Report on Consultation. Quality and Fairness - A Health System for you.

[26] Darker CD, Donnelly-Swift E, Whiston L (2018). Demographic factors and attitudes that influence the support of the general public for the introduction of universal healthcare in Ireland: a national survey. Health Policy.

[27] Thomas S, O’Donoghue C, McCarthy N, Almirall-Sanchez A, Burke S, Keegan C, Dempsey G, Barry S, Fleming P. The PRESTO report. 2023.

[28] WHO. Universal Health Coverage (UHC) – fact sheet. WHO; 2023. https://www.who.int/news-room/fact-sheets/detail/universal-health-coverage-(uhc).

[29] Tussing DA, Wren MA (2005). How Ireland cares. The case for healthcare reform.

[30] Houses of the Oireachtas Committee on the Future of Healthcare (2017). Houses of the Oireachtas Committee on the future of Healthcare Sláintecare Report, May 2017.

[31] Department of Health (2018). Slaintecare implementation strategy.

[32] Thomas S, Johnston B, Barry S, Siersbaek R, Burke S. Sláintecare implementation status in 2020: limited progress with entitlement expansion. Health Policy 2021. 10.1016/j.healthpol.2021.01.009 PMC9757858 33531170

[33] Burke S, Johnston B, Thomas S. Houses of the Oireachtas : Opening Statement to Oirehactas Committee on Health on issues relating to the implementation of Sláintecare reforms. (Health HotOCo ed. https://data.oireachtas.ie/ie/oireachtas/committee/dail/33/joint_committee_on_health/submissions/2023/2023-03-01_opening-statement-dr-sara-burke-associate-professor-in-health-policy-and-management-centre-for-health-policy-and-management-trinity-college-dublin_en.pdf: 2023.

[34] Burke S, Parker S, Fleming P, Barry S, Thomas S (2021). Building health system resilience through policy development in response to COVID-19 in Ireland: from shock to reform. Lancet Reg Health - Europe.

[35] Parker S, Mac Conghail L, Siersbaek R, Burke S. How to not revert to type; complexity-informed Learnings from the pandemic response for Health System Reform and Universal Access to Integrated Care. Front Public Health 2023, 11. 10.3389/fpubh.2023.1088728 PMC9996344 36908402

[36] Johnston B, Burke S, Kavanagh P, O’Sullivan C, Thomas S, Parker S. Moving beyond formulae: a review of international population-based resource allocation policy and implications for Ireland in an era of healthcare reform [version 1; peer review: 1 approved]. HRB Open Res 2021, 4.

[37] Department of Health. Sláintecare Implementation Strategy & Action Plan 2021–2023. https://www.gov.ie/en/publication/6996b-slaintecare-implementation-strategy-and-action-plan-2021-2023/: Department of Health; 2021.

[38] Go IHSE. Organisational Reform. HSE Health regions. Government of Ireland; 2023. https://www.gov.ie/en/publication/4eda4-slaintecare-regional-health-areas-rhas/#hse-health-regions-implementation-plan.

[39] Burke S, Thomas S, Stach M, Kavanagh P, Magahy L, Johnston B, Barry S. Health system foundations for Sláintecare implementation in 2020 and beyond ? Co-producing a Sláintecare living implementation Framework with evaluation: learning from the Irish health system?s response to COVID-19. A mixed-methods study protocol [version 1; peer review: awaiting peer review]. HRB Open Res 2020, 3. 10.12688/hrbopenres.13150.1 PMC7934093 33728398

[40] website TFt. Foundations https://www.tcd.ie/medicine/health-policy-and-management/research/current-projects/foundations/: Trinity 2023.

[41] Marron L, Burke S, Kavanagh P. Changes in the utilisation of acute hospital care in Ireland during the first wave of the COVID-19 pandemic in 2020 [version 3; peer review: 2 approved, 1 approved with reservations]. HRB Open Res 2022, 4. 10.12688/hrbopenres.13307.1 PMC9513415 36204710

[42] McGlacken-Byrne D, Parker S, Burke S. Tracking aspects of healthcare activity during the first nine months of COVID-19 in Ireland: a secondary analysis of publicly available data [version 2; peer review: 2 approved with reservations]. HRB Open Res 2023, 4. 10.12688/hrbopenres.13372.3 PMC11439124 39347503

[43] Parker S, Siersbaek R, Mac Conghail L, Burke S (2023). Public Health Responses to Homelessness during COVID-19 in Ireland: implications for Health Reform. Int J Homelessness.

[44] Asdal K, Reinertsen H. Doing document analysis: a practice-oriented method. Sage; 2022.

[45] S BSK (2014). InterViews: learning the craft of qualitative research interviewing.

[46] Braun V, Clarke V. Thematic analysis. A practical guide. Sage; 2021.

[47] Government of Ireland. : An Taoiseach and Minister Harris announce Executive Director of Sláintecare Programme Office and Chair of Advisory Council to lead health reform. https://www.gov.ie/en/press-release/564551-an-taoiseach-and-minister-harris-announce-executive-director-of-slai/: Government of Ireland; 2018.

[48] Department of Health. Sláintecare Action Plan 2019. Department of Health (DoH); 2019.

[49] Department of Health (2019). Minister for Health confirms restructuring of health services and delivers key Sláintecare commitment. Questions & answers. (Health do ed.

[50] Wall M. Magahy cited slow progress in key areas of reform in resignation letter. In *The Irish Times*. Dublin: https://www.irishtimes.com/news/ireland/irish-news/magahy-cited-slow-progress-in-key-areas-of-reform-in-resignation-letter-1.4677203; 2018.

[51] O’Connell H. HSE boss and top civil servant to co-chair new Sláintecare board. Irish independent. Irish independent; 2021. https://www.independent.ie/irish-news/hse-boss-and-top-civil-servant-to-co-chair-new-slaintecare-board/40921082.html.

[52] Mindo, Minister Donnelly announces establishment of Regional Health Areas Advisory Group. In. Medical independent. Medical independent; 2021. https://www.medicalindependent.ie/in-the-news/breaking-news/minister-donnelly-announces-establishment-of-regional-health-areas-advisory-group/.

[53] Department of Health. Business Case for the Implementation of Regional Health Areas (RHAs). https://assets.gov.ie/220582/178975ac-74de-40ee-8131-37db61c64612.pdf: Department of Health 2022.

[54] Department of Health. Sláintecare Action Plan 2022. Department of Health; 2022. https://www.gov.ie/en/publication/0d2d60-slaintecare-publications/#slaintecare-action-plan-2022.

[55] Quigley J, Coyle C, O’Dwyer C, O’Brien D, O’Nolan G, Farragher L, Long J (2019). Regional health organisations: an evidence review.

[56] Vrangbaek K. Towards a typology for decentralization in health care. In *Decentralization in health care: Strategies and Outcomes* Edited by Saltman RB, Bankauskaite V, Vrangbaek K. Berkshire, England: Open University Press; 2007: 44–62.

[57] Mather J. Transparency in the European Union. *Openness and transparency: meaningful or meaningless? Access to information on the European Union Manchester: European Information Association 1997* Publications Office, European, Commission – Information. Communication Culture Audiovisual, Media; 1996.

[58] Department of Health. Sláintecare Action Plan 2023. Department of Health; 2023. https://www.gov.ie/en/publication/0d2d60-slaintecare-publications/#slaintecare-action-plan-2023.

[59] World Health Organization. Monitoring the building blocks of health systems: a handbook of indicators and their measurement strategies. World Health Organization; 2010.

[60] Bruen C, Brugha R, Kageni A, Wafula F (2014). A concept in flux: questioning accountability in the context of global health cooperation. Globalization Health.

[61] Government of Ireland. Government to establish new independent Board for the HSE. Government of Ireland; 2018. https://merrionstreet.ie/en/news-room/news/government_to_establish_new_independent_board_for_the_hse.html.

[62] Lipsitz LA (2012). Understanding Health Care as a Complex System: the Foundation for Unintended consequences. JAMA.

[63] Department of Health and Children (2003). Commission on Financial Management and Control Systems in the Health Service (Brennan Report).

[64] Department of Health and Children (2003). Audit and structures and functions in the Health System (the Prospectus Report).

[65] Department of Health and Children (2010). Report of the Expert Group on Resource Allocation and Financing in the Health Sector.

[66] Murphy D. Donnelly left ‘hang out to dry’ over health Budget, says Sinn Féin. RTE; 2023. https://www.rte.ie/news/politics/2023/1012/1410564-politics-leaders-questions/.

[67] Johnson H. All vision but no change? Determinants of implementation. The Case of Ireland and Mental Health Policy. In.; 2015.

[68] Daly F, Edwards C. Tracing state accountability for COVID-19: representing care within Ireland’s response to the pandemic. Social Policy Soc 2022:1–13.

[69] Clay EJ, Schaffer B (1984). Room for Manoeuvre: An Exploration of Public Policy Planning in Agricultural and Rural Development.

[70] Eckl J, Hanrieder T (2023). The political economy of consulting firms in reform processes: the case of the World Health Organization. Rev Int Polit Econ.

[71] Sheard S (2018). History matters: the Critical Contribution of Historical Analysis to Contemporary Health Policy and Health Care. Health Care Anal.

[72] Wren MA (2003). Unhealthy state. Anantomy of a Sick Society.

[73] Burke S. *Irish Apartheid. Healthcare Inequality in Ireland* 1st edition edn. Dublin: New Island; 2009.

[74] Metz A, Jensen T, Farley A, Boaz A, Bartley L, Villodas M. Building trusting relationships to support implementation: a proposed theoretical model. Front Health Serv 2022, 2. 10.3389/frhs.2022.894599 PMC10012819 36925800

[75] McKee CM, Greenley. WHO/European Observatory on Health Systems and Polices, Rachel, Permanand,: Trust. The foundation of health systems In *Policy brief 58* (Lessof S, Azzopardi Muscat, N, Permanand, G, Figueras, J. ed. https://eurohealthobservatory.who.int/publications/i/trust-the-foundation-of-health-systems: 2023. 39585948

