# Supplementary material for: The role of governance in shaping health system reform: a case study of the design and implementation of new health regions in Ireland, 2018–2023
Source: BMC Health Serv Res. 2024 May 3;24:578. doi: 10.1186/s12913-024-11048-2 (PMC11069256; doi:10.1186/s12913-024-11048-2)
Supplement: Supplementary file 2 — Supplementary Material 2 [file 12913_2024_11048_MOESM2_ESM.docx]

**INTERVIEW GUIDE**

**Foundations Project – Role of Governance in Health Regions Reform**

**QUICK Opening Question (5 mins)**

1. **Can you briefly tell me about your role and how you were/are involved in the design and/or implementation of the regions?**

We are particularly interested in finding out more about the decisions that have influenced implementation; as well the governance of the implementation of the regions since 2017. By governance we mean the processes and institutions through which decisions are made and acted upon.

1. **From your perspective, which actors** (people/units/ organisations) **have had/have responsibility for implementing the regions pre COVID and now since 2021?**
2. Was/Is it clear who was/is responsible for what, and who they report/ed to?
3. What were/are the key decisions which have or are influencing the implementation of the regions? **(10 mins)**

1. **Policy capacity** What resources did/do senior policy makers have to facilitate implementation of the regions? E.g. intelligence and evidence production, specialist advice, internal staff capacity, appropriate budget?

1. **Participation**: How participative wa/is the implementation process? Eg consultation, stakeholder forums, committee membership, Are/Were national or local elected representatives, those on the frontline, patients or members of the public involved, and if so how?

1. **Transparency**: How do those responsible for implementing the regions keep key stakeholders informed of key decisions and progress? Eg committees, reporting, assessment of progress. *How could this have been/be improved?*

1. **Accountability:** Were or are there mechanisms in place to ensure key decision-makers progress implementation and are held to account for progress/lack of progress? E.g. implementation goals and targets, reporting to oversight committees, outside agencies. Were/Is there any consequences for not progressing implementation of the regions?

1. **Integrity:** Are/Were there clear oversight mechanisms for overseeing implementing agents or managing potential conflicts of interest? Or conflicting policy advice?

1. **What do you think are the three key lessons from regional implementation to date?**
2. **Do you think we should have regions, do you think there will be regions?**
3. **Is there anything else you want to tell me? Anyone else I should interview?**
